# The Relationship Between Fiber Intake and Gut Bacterial Diversity and Composition During the Third Trimester of Pregnancy

**DOI:** 10.3390/nu17050773

**Published:** 2025-02-22

**Authors:** Lindsay T. Schwartz, Jillian G. Ladouceur, Madeleine M. Russell, Shiyi Y. L. Xie, Sihan Bu, Jean M. Kerver, Sarah S. Comstock

**Affiliations:** 1 Department of Food Science and Human Nutrition, Michigan State University, East Lansing, MI 48824, USA; 2 Department of Epidemiology and Biostatistics, Michigan State University, East Lansing, MI 48824, USA

**Keywords:** pregnancy, nutrition, fiber, microbiota, microbiome, bacteria, butyrate, diet

## Abstract

Background/Objectives: High fiber (34–36 g/day) diets are recommended during pregnancy due to inverse associations with constipation and adverse pregnancy health outcomes, including pre-eclampsia and gestational diabetes. However, the mechanism for this protective effect is poorly defined. Fiber may be protective due to its impact on the composition and function of specific bacteria within the pregnancy gut microbiome. The purpose of this analysis was to investigate whether a sub-sample of cohort study participants in their third trimester met daily dietary fiber and vegetable intake recommendations and, in turn, how this impacted bacterial composition and butyrate-producing genes within the gut microbiome. Methods: Pregnant participants (*n* = 52) provided stool samples and survey data, which were used to calculate fiber and vegetable intake. Genomic DNA was extracted from the stool samples, followed by PCR to amplify the V4 region of the 16S rRNA gene. Amplicons were sequenced and mapped to the RDP reference. Quantitative real-time PCR was used to measure the abundance of bacterial genes for butyrate production. Results: Of the pregnant participants in this sample, 84.7% and 92.3% failed to meet recommendations in the Dietary Guidelines for Americans for dietary fiber and vegetable intake, respectively. All four participants who met the vegetable recommendation were a subset of those who met the fiber recommendation. The participants who met the pregnancy fiber recommendation had gut microbiotas with greater alpha diversity (Shannon and Inverse Simpson) than those who did not. However, there was no association between dietary fiber intake and the abundance of bacterial genes for butyrate production. Conclusions: This research suggests that general fiber intake during pregnancy has a modest association with the gut bacterial community. These preliminary results demonstrate a need to improve fiber intake during pregnancy. Further, studies that measure the relationship between dietary intake of specific types of fiber and associations with specific gut bacterial community members and their functions are needed.

## 1. Introduction

A diet rich in dietary fiber has been associated with numerous health-promoting factors for pregnant women. A high intake of dietary fiber has been shown to increase gut microbiome diversity [1,2]. Extending beyond the effects on the microbiome, dietary fiber reduces intestinal transit time, improves metabolic activity, and increases the frequency of defecation [3]. Additionally, high fiber intake reduces the risk for and improves the management of various diseases [4,5]. During pregnancy, increased levels of progesterone can increase the risk of constipation, and increased consumption of dietary fiber has been shown to reduce this risk [6]. Additionally, dietary fiber has been demonstrated to help pregnant women achieve appropriate gestational weight gain, reduce the risk of pre-eclampsia, and reduce the risk of gestational diabetes [6,7]. Decreased fiber intake during pregnancy has been associated with cognitive delays in children [8]. Dietary intake data from the National Health and Nutrition Examination Survey Research (NHANES) shows that the majority of Americans do not meet the recommended fiber intake levels, with pregnant women on average consuming 17.3 g of fiber/day [9], nearly 15 g/day below the recommendation. Thus, increasing fiber intake among pregnant women is a priority [6,10,11].

The Dietary Guidelines for Americans 2020–2025 include dietary recommendations for pregnancy [12]. Women in their third trimester of pregnancy, aged 14–18 y or 31–50 y, are advised to consume 34 g of fiber, while women aged 19–30 y are advised to consume a slightly higher amount, at 36 g of fiber/day. These guidelines are based on intake levels shown to lower the risk of coronary heart disease. Thus, the average US consumption of ~17 g/day indicates that most pregnant women consume amounts of fiber that are vastly lower than the recommended amounts. Some studies indicate that the average dietary fiber intake of women, both non-pregnant and pregnant, is below the recommended guidelines. The average dietary fiber for a woman aged 19–30 and 31–50 years is 13.5 g/day and 14.1 g/day, respectively, which is below the Adequate Intake (AI) recommendation of 25 g per day [13]. In one study, the average fiber intake for participants during the last month of pregnancy was 15.0 g/day, which is lower than the DGA recommended intake of 34 g/day, and none of the participants met the recommended guideline [14]. In another study, the average fiber intake among study participants was 18.6 ± 3.8 g/day, which is significantly lower than the 2020–2025 DGA recommended intake of 31–36 g/day, and none of the participants met the recommended guidelines [15]. Vegetables are another important source of fiber to be consumed in sufficient amounts during pregnancy. The Dietary Guidelines for Americans 2020–2025 states that pregnant women should consume between 2.5 and 3.5 cups of vegetables per day depending on total caloric intake. These guidelines formed the basis to assess the adequacy of fiber and vegetable intake of the pregnant participants in this study.

Fiber is fermented by bacteria in the colon. Plant-rich and high-fiber diets have been positively associated with an increased abundance of specific bacterial taxa, including *Ruminococcus* and *Veillonella* [16]. Decreased dietary fiber has been associated with an increased abundance of bacterial taxa such as *Collinsella*, which was further linked to hyperinsulinemia [17,18]. Bacterial metabolic processes produce numerous metabolites, such as butyrate, a short-chain fatty acid. Butyrate serves as a major energy source for colonocytes and is crucial for a healthy colon [19]. In addition to being a major energy source for these cells, butyrate prevents intestinal and extraintestinal diseases as well as improves overall health [20,21]. Butyrate can protect against such diseases due to its anti-inflammatory properties and its ability to regulate gene expression [21]. Herein, we measure the relative abundance of the final genes in two main bacterial butyrate synthesis pathways: butyryl-CoA–acetate CoA-transferase (*but*) and butyrate kinase (*buk*) [19]. We hypothesized that participants who consumed an amount of dietary fiber above the recommendation would have a higher abundance of these butyrate production genes than participants who did not meet the fiber recommendation.

Specific associations between dietary fiber intake, vegetable intake, and the gut microbiota composition, diversity, and butyrate production capacity have been poorly described. Thus, there were three main objectives for this study: (1) to determine whether the study participants in a Michigan cohort meet US recommendations for dietary fiber and vegetable intake in their third trimester of pregnancy, (2) to determine if there is a significant association between fiber intake that met the recommendation and gut bacteria during the third trimester of pregnancy, and (3) to measure associations between dietary fiber intake and the *but* and *buk* bacterial genes for butyrate production. We hypothesized that the gut microbiota of the pregnant participants who consumed fiber at or above the recommended amount would have a greater abundance of health-promoting bacterial taxa compared to those who did not meet the recommendation for fiber intake.

## 2. Methods

### 2.1. Study Population and Demographics

**Figure 1:**
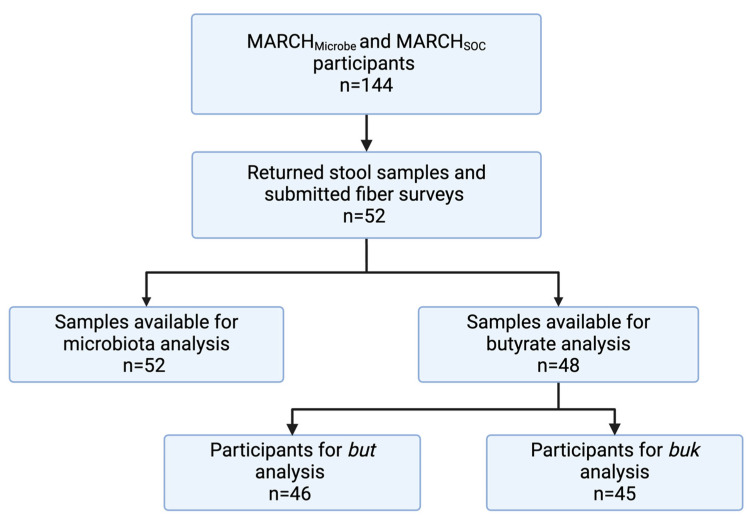
Participant flow chart.

The study participants (*n* = 52) for this analysis were recruited from a prospective pregnancy cohort—the Michigan Archive for Research on Child Health (MARCH) (Figure 1). The participants were enrolled in the MARCH (IRB#16-1429) parent study, and a subset of participants consented to participate in MARCH MICROBE (IRB #17-1352) or MARCH Stress of COVID-19 (SOC) (IRB#00005020), which are sub-studies of the parent MARCH cohort. The participants in the two sub-studies provided stool samples and reported fiber intake during pregnancy. There were 52 samples available for microbiota analysis.

The pregnant participants included in this study were in their third trimester of pregnancy and ranged from 20 to 53 years of age. The exclusion criteria included age <18 y at enrollment and the inability to communicate with study staff in the English language. All 52 participants were included in the dietary fiber and microbiota analysis. A smaller sample size (*n* = 48) was available for butyrate-producing gene analysis due to a lack of sufficient stool sample or when the two cycle threshold values for replicate assay of the same sample varied by more than one PCR cycle. Samples from 46 of the participants were included for *but* gene analysis, and samples from 45 of the participants were included for *buk* gene analysis. For these studies, all participants gave written informed consent. Participant demographic data, such as education level and household income, were obtained via prenatal intake forms. Further participant characteristics, such as age, race, pre-pregnancy BMI, and if the participant had ever smoked, were obtained via their infant’s birth certificate data. Participants were grouped into pre-pregnancy BMI categories based on the Centers for Disease Control (CDC) recommendations as normal (18.5 < BMI < 25; *n* = 25), overweight (25 ≤ BMI ≤ 30; *n* = 9), and obese (BMI > 30, *n* = 13).

### 2.2. Dietary Assessment and Data Collection

All participants completed a National Cancer Institute (NCI) five-factor screener during their third trimester of pregnancy [22,23]. This is a validated survey that asks about the frequency of intake for a variety of food groups and beverages. This survey did not ask about the portion sizes consumed by each participant, but the equations used to calculate fiber intake account for average portion sizes based on age and biological sex. Data from the surveys were collected and cleaned for analysis. Fecal samples were collected by participants using methods previously described [24], with the sample placed in an OMNIgene-GUT tube (DNA Genotek Inc., Stittsville, ON, Canada) and shaken in order to preserve DNA during shipping. Samples were returned to the lab by US mail, aliquoted, and stored in a −80 °C freezer.

### 2.3. DNA Extraction and Gene Sequencing

All methods were conducted as described in [25]. Briefly, genomic DNA (gDNA) was extracted from fecal samples using the DNeasy PowerSoil Pro DNA Isolation Kit (Qiagen, Carlsbad, CA, USA). The concentration was determined using the Nanodrop Spectrophotometer (ThermoFisher Scientific, Madison, WI, USA). The V4 region of the 16S rRNA gene was amplified using Schloss lab primers [26], AccuPrime Pfx SuperMix (ThermoFisher Scientific, Waltham, MA, USA), and template DNA. The PCR reactions were performed in triplicate. The PCR amplicons were verified using agarose gel electrophoresis. Once it was confirmed that the amplicons were copied successfully, the triplicate amplicons were pooled and cleaned using Agencourt AMPure XP beads for PCR purification (Beckman Coulter, Brea, CA, USA). The resulting amplicon libraries were submitted for 16S rRNA gene sequencing. The Michigan State University Genomics Research Technology Support Facility completed the sequencing using an Illumina MiSeq (Illumina, Inc., San Diego, CA, USA) and 250 bp paired-end sequencing with V2 chemistry.

### 2.4. Sequencing Data Processing

The 16S rRNA gene sequences were processed using mothur. The MiSeq standard operating procedure was followed for processing [27]. Reads were rarefied to 15,000 reads per sample before further analysis was performed. Rarefaction curves were created to ensure sufficient community coverage. Taxonomy was assigned using operational taxonomy units according to phylotype using version 18 of the RDP reference database.

### 2.5. Butyrate Production Gene Measurement

As previously described [28], quantitative real-time PCR (qRT-PCR) was performed using genomic DNA from each sample to determine the levels of butyryl-CoA–acetate CoA-transferase (*but*) and butyrate kinase (*buk*). Primer sets, But_F.prausn, and Buk, were utilized as previously described [19]. For the purposes of this assay, DNA was quantified using a quant-iT dsDNA Assay Kit, High Sensitivity (Thermo Scientific REF Q33120). All genomic DNA samples were diluted to 2.5 ng/μL. All wells in a 96-well plate were filled with 7.5 μL SYBR Green, 3.5 μL ddH_2_O, 1 μL F primer, 1 μL R Primer, and 2 μL template. The thermocycling program was as follows: 2 min at 50 °C, 10 min at 95 °C, 45 s at 95 °C, 45 s at annealing temperature for the specific primer (70 °C for *but* or 64 °C for *buk*), and 45 s at 72 °C, with the sequence of the three 45 s steps repeated for 40 cycles. DNA was amplified in a QuantStudio 7-plex (Applied Biosystems, Waltham, MA, USA). For each gene, all samples were run on the same sample plate, and cycle threshold values were used for analysis.

### 2.6. Statistical Analysis

Participant characteristics were compared between those who met the fiber recommendation and those who did not meet the fiber recommendation using a chi-square test of proportions. PhenX protocol equations were used to calculate fiber and vegetable intake from the NCI Five-Factor Screener responses [22]. The vegan package in R, with the RStudio interface, was used to analyze data for alpha and beta diversity, as previously described [25]. Alpha diversity is the bacterial diversity within a single community. Chao 1, Inverse Simpson, and Shannon indices of alpha diversity were calculated using the vegan package in R. Chao 1 measures richness—the presence or absence of bacteria. Shannon measures abundance with an even weight for all bacteria, while inverse Simpson measures abundance, putting more weight on abundant bacteria. The Shapiro–Wilk test was used to determine if the data were normally distributed. When the data were normally distributed, *t*-tests were used to compare alpha diversity between those who met the recommendation versus those who did not. When the data were not normally distributed, Wilcoxon rank sum tests were used to compare microbial alpha diversity.

Beta diversity measures the similarity in bacteria between two communities, with Sorensen measuring richness and Bray–Curtis measuring evenness. Beta diversity was visualized via a principal coordinate analysis (PCoA). Beta diversity was assessed by PERMANOVA (adonis2, vegan 2.6-4 R package), and sample dispersion was measured via PERMDISP (betadisper) using the vegan package in R. PERMANOVA compares centroids of each group, while PERMDISP tests for differences in the variance in the scatter within each group. Bacteria with a mean relative abundance greater than or equal to 1% were included in Bray–Curtis PCoA plots that show the correlation between bacterial abundance and the plot coordinates, where arrows show directionality between significant taxa and increased abundance. For the negative binomial analysis, taxa at the genus level were included that had a mean relative abundance greater than or equal to 1%. Median count values are reported. *p*-values were adjusted for the false discovery rate with Benjamini–Hochberg correction. Significance was set to *p* less than 0.05.

To quantify the relative abundance of the *but* and *buk* genes for butyrate production, a quantitative, real-time polymerase chain reaction was performed [28]. A greater cycle threshold (CT) value indicated a lower relative abundance of the target gene and, as such, reduced butyrate production capacity. The Shapiro–Wilk normality test was used to test if the data were normally distributed. After performing the Shapiro–Wilk test on the *but* and *buk* CT values, the data were determined to be non-parametric. Wilcoxon rank sum tests were conducted to determine whether there was a significant relationship between dietary fiber intake and CT values of each butyrate-producing bacterial gene. *p*-values less than 0.05 were considered significant for all tests.

## 3. Results

### 3.1. Participant Demographics

Apart from age, participant characteristics were similar for those who met and those who did not meet the fiber recommendation. The analyzed characteristics included maternal age, BMI category, race, history of smoking, annual household income, and education level (Table 1). Data were missing for some of these variables for some participants (noted as a footnote to the table).

### 3.2. Pregnancy Dietary Fiber Intake

**Figure 2:**
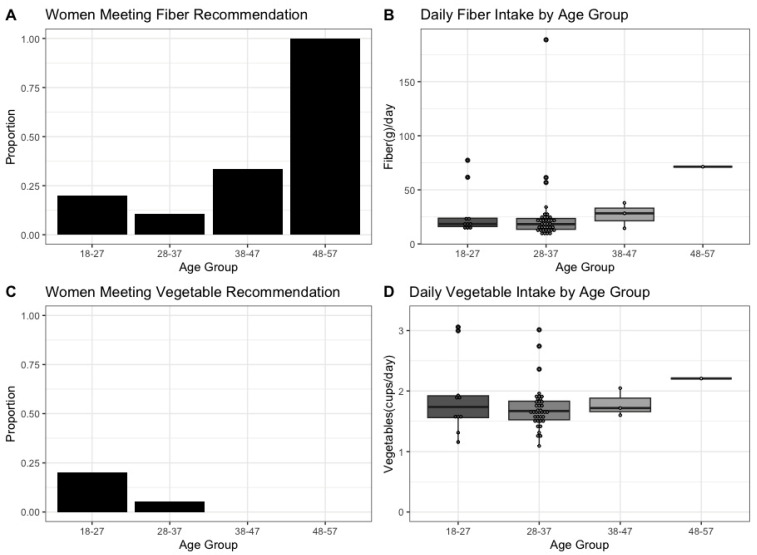
Fiber and vegetable intake during pregnancy by age group. Proportion of participants in each age group meeting the dietary fiber recommendation (**A**). Daily fiber intake by age group (**B**). The fiber recommendation during pregnancy is 34–36 g/fiber per day. Proportion of participants in each age group meeting vegetable intake recommendations (**C**). Daily vegetable intake by age group (**D**). The vegetable recommendation during pregnancy is 2.5 cups of vegetables/per day. The line across the boxplot indicates the median, the whiskers of the boxplot represent the range, and the inner portion of the boxplot is the interquartile range. Since only one individual was represented in the 48–57 age group, the boxplot is represented as a straight line.

In the 18–27 year age group (*n* = 10), 20% of the participants consumed an amount of dietary fiber at or above the third-trimester recommendation, whereas 10.5% of the participants in the 28–37 year age group (*n* = 38), 33% of the participants in the 38–47 year age group (*n* = 3), and 100% of the participants in the 48–57 age group (*n* = 1) met the recommendation (Figure 2A). Only 8 of the 52 total participants met the fiber recommendation. The women between 18 and 27 years of age consumed a median of 18.4 g/day (Figure 2B). The women between 28 and 37 years of age consumed a median of 18.3 g/day. Those in the 38–47 year age group consumed a median of 28.3 g/day, and those in the 48–57 years age group consumed a median of 71.4 g/day. Note that the 48–57 year age group included a single participant. The overall median fiber intake was 18.6 g per day, which is below the recommendation of 34 to 36 g/day for pregnant women described in the Dietary Guidelines for Americans 2020–2025 [12].

### 3.3. Pregnancy Vegetable Intake

The percentage of women who met the vegetable recommendation was analyzed by age group (Figure 2C). Only 4 of the 52 participants met the vegetable recommendation. This was a subset of those who met the fiber recommendation. Overall, 20 percent of the participants in the 18–27 age group, 5.3% of the participants in the 28–37 age group, 0% of the participants in the 38–47 age group, and 0% of the participants in the 48–57 age group consumed a quantity of vegetables at or above the third-trimester recommendation. The women between 18 and 27 years of age consumed a median of 1.72 cups/day (Figure 2D), and the women between 28 and 37 years of age consumed a median of 1.65 cups/day. Those in the 38–47 year age group consumed a median of 1.7 cups/day, and those in the 48–57 year age group consumed a median of 2.19 cups/day. The overall median vegetable intake was 1.65 cups of vegetables per day, which is below the recommendation of 2.5 to 3.5 cups/day for pregnant women described in the Dietary Guidelines for Americans 2020–2025 [12].

### 3.4. Diversity of the Pregnancy Gut Microbiota

**Figure 3:**
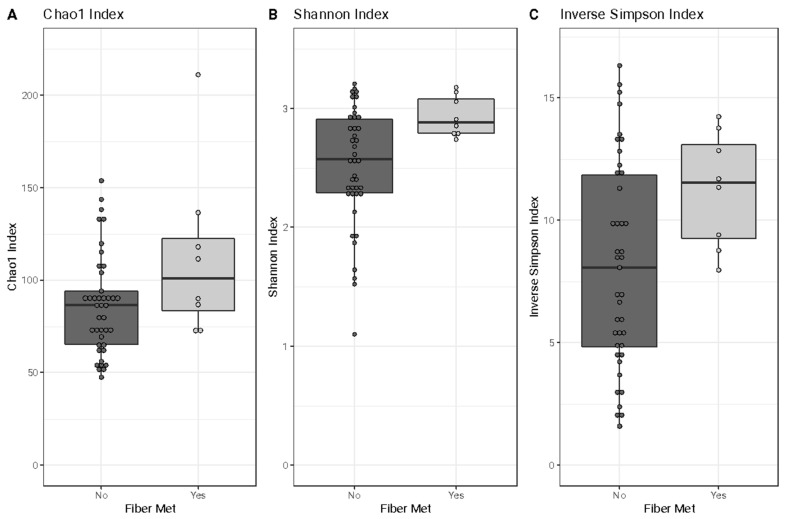
Box-and-whisker plots of Chao1 (**A**), Shannon (**B**), and Inverse Simpson alpha diversity indices (**C**) by whether women met (Yes, *n* = 8) or did not meet (No, *n* = 44) the fiber recommendation. The line across the boxplot indicates the median, the whiskers of the boxplot represent the range, and the inner portion of the boxplot is the interquartile range. The participants who met the fiber recommendation during pregnancy had gut microbiotas with similar richness (Chao1, *p*-value = 0.14) but greater microbial diversity (Shannon, *p*-value = 0.02; Inverse Simpson, *p*-value < 0.01) compared to the participants who did not meet the fiber recommendation.

For within-sample analysis, there was no difference in richness (Figure 3A) between the women who met their dietary fiber recommendation and those who did not (Chao 1, *p*-value > 0.05). However, the women who met their daily dietary fiber recommendation had gut microbiotas with increased bacterial diversity (Shannon and Inverse Simpson, *p*-value < 0.05) relative to the women who did not (Figure 3B,C).

**Figure 4:**
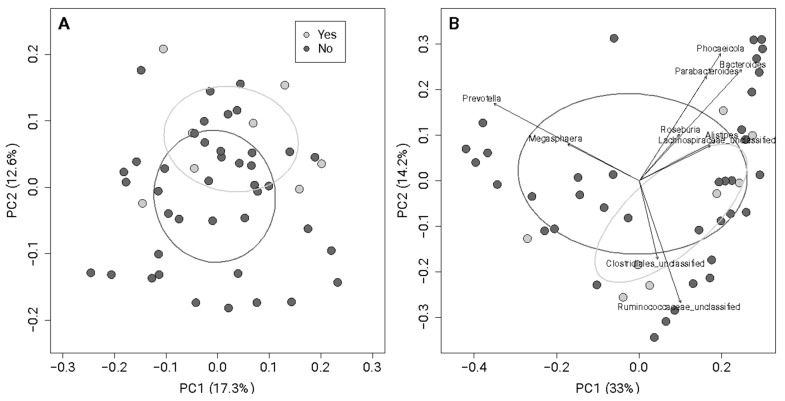
PCoA of Sorensen (**A**) and Bray–Curtis (**B**) dissimilarity with associated genera. Women who met the fiber recommendation intake (Yes, light grey) had similar gut microbial communities to those who did not (No, dark grey) based on composition ((**A**), Sorensen, *p*-value = 0.54) and community structure ((**B**), Bray–Curtis, *p*-value = 0.50). Each dot represents an individual participant, and the ellipses are based on the centroids of each group. The further apart the dots, the greater the dissimilarity. The x-axis represents the percentage of variance explained by PCoA1, and the y-axis represents the percentage of variation explained by PCoA2.

When comparing gut bacterial community membership (Figure 4A) and composition (Figure 4B), there were no significant differences in the gut bacterial communities of the women who met their daily fiber recommendation and those who did not (Sorensen and Bray–Curtis, PERMANOVA *p*-value > 0.05). Based on the distinct localization of the participants with high abundances of Bacteroides, Prevotella, or Ruminococcus on the PCoA plot (Figure 4B), our data indicate that the gut microbiomes of the pregnant participants seemingly fell into three common enterotypes: *Bacteroides*, *Prevotella*, and *Ruminococcus* [2,29].

Following this, a negative binomial analysis, inclusive of taxa with an average abundance greater than or equal to 1% of the relative abundance, revealed a significant difference in the abundance of *Prevotellamassilia* and *Phascolarctobacterium* within the women who met the dietary fiber recommendation (Supplemental Table S1, *p*-value < 0.0001). The women who failed to meet the fiber recommendation had a lower number of those bacterial taxa than those who met the dietary fiber recommendation. Due to the small number of women who met the recommendation, these taxa-level differences should be considered exploratory results.

Due to the low sample size and the overlap in the participants who met/did not meet the fiber recommendation, we did not compare microbiota characteristics for meeting versus not meeting the vegetable recommendation.

### 3.5. Butyrate Production Capacity of the Maternal Gut Microbiota

**Figure 5:**
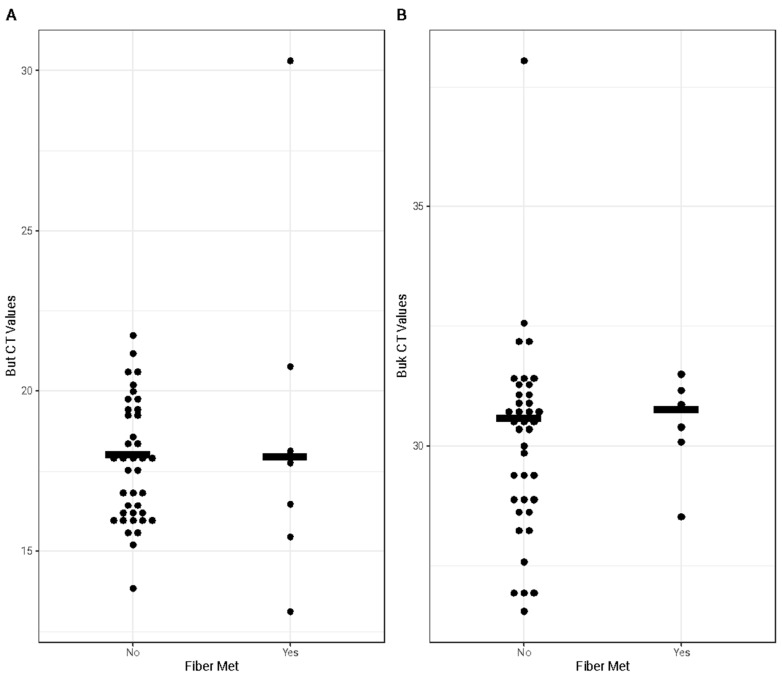
Bacterial butyrate-producing genes. CT values of the but (**A**) and buk (**B**) genes based on meeting (Yes) versus not meeting (No) the fiber recommendation. Each black dot represents a participant. The solid black lines represent the median CT value of each group. There were no significant differences in the abundance of the but (*p*-value = 0.29) or buk (*p*-value = 0.61) genes detected in either group.

Butyrate production capacity was similar whether a pregnant woman met or did not meet the fiber intake recommendation. The abundance of *but* (Figure 5A) and *buk* (Figure 5B) genes are depicted by cycle threshold (CT) values, where a low CT value corresponds to a high quantity of the respective butyrate-producing gene. The abundance of the *but* gene (*p*-value = 0.29) and of the *buk* gene (*p*-value = 0.61) in the gut microbiota was similar for the women who met and did not meet the recommendation.

## 4. Discussion

The median fiber consumption for women in this cohort was 18.6 g of fiber per day, which is below the recommended pregnancy fiber intake of about 35 g/day (depending on age group). With only 8 out of the 52 women meeting their fiber recommendation, most of the pregnant women in our study consumed well below the recommended amount of dietary fiber put in place by the Dietary Guidelines for Americans 2020–2025 [12]. Furthermore, only four of the participants in our study met the vegetable recommendation. Not surprisingly, since vegetables are a good source of fiber, the women who met the vegetable recommendation were a subset of the women who met the dietary fiber recommendation. The majority of our participants consumed an amount of dietary fiber that is below the recommendation. Other studies that analyzed dietary fiber intake in pregnant women concluded that pregnant women did not consume an adequate amount of fiber [6,10,11]. Thus, the results of this study are in line with those of prior reports.

Women who met their fiber recommendation had a higher within-sample abundance and evenness relative to those who did not meet their recommendation. Within previous intervention studies, pregnant women who consumed high fiber showed significant differences in changes in observed species compared to those with typical fiber intake [30]. Increased species richness and diversity have also been observed over time with higher dietary fiber intake in pregnant women [31]. However, another study found no association between fiber intake and alpha diversity measures [18]. We found no difference in the community structure and composition between the women who met their fiber intake and those who did not; however, we did find that the participants’ gut microbiotas grouped into three common enterotypes, i.e., *Bacteroides*, *Prevotella*, and *Ruminococcus*, based on taxa-level associations (Figure 4B).

Additionally, taxa-level differences have been observed within the microbiomes of those consuming high fiber during pregnancy, including decreased *Collinsella* [17,18,32], which was not among the taxa present at >1% abundance in the gut microbiotas of the pregnant women in our study. Several studies have demonstrated the impact of *Collinsella* on health conditions; low dietary fiber intake may lead to the overgrowth of *Collinsella*, which is positively correlated with increased insulin levels [18]. Patients with mixed irritable bowel syndrome (IBS-M) have an increased abundance of *Collinsella aerofaciens* [33]. *Collinsella* is positively correlated with fasting levels of triglycerides and total cholesterol and negatively correlated with high-density lipoprotein (HDL) cholesterol [34]. Common taxa that would normally be associated with a healthy gut, such as *Prevotella*, were present at similar abundance in the groups of women who met their fiber recommendation and in those who did not meet their recommendation. This differed from our initial hypothesis, as a high abundance of *Prevotella* typically is associated with a high fiber diet [35,36].

Within our negative binomial analysis, we also observed that the women who met their fiber recommendation had an increased abundance of *Prevotellamassilia* and *Phascolarctobacterium*. *Prevotellamassilia* has been associated with being anti-inflammatory and as having butyrate-producing potential within the gut microbiome [37]. While *Prevotellamassilia* was found to be greater in the women who met their fiber recommendation, this was driven by 1–2 individuals with a high abundance of this specific taxa. Thus, though fiber may play a role in the abundance of *Prevotellamassilia*, we cannot conclusively say that fiber intake positively correlated with *Prevotellamassilia* abundance. Additionally, another study investigating the gut microbiome and metabolites in patients with type 2 diabetes mellitus detected enriched *Phascolarctobacterium* in the microbiome of healthy controls, and the increased abundance of this taxa was associated with increased absorption of alpha-linolenic acid [38]. Together, these results begin to demonstrate a relationship between dietary fiber and the complex downstream associations with specific taxa. However, larger studies with a greater proportion of women meeting their fiber recommendation are necessary to understand the taxa-level changes associated with increased fiber. Additionally, larger sample sizes are needed to understand how specific types of fiber drive taxa abundance, as demonstrated by the results of a previous study, which highlight how the diversity of fiber structure drives microbial composition of the gut microbiome [39]. Additionally, this group demonstrated that phylogenetic diversity and high fiber intake drive microbial composition towards increased obligate anaerobes, decreasing inflammation and antimicrobial resistance [40].

We observed no significant differences between the *but*/*buk* gene abundances for butyrate production between the women who consumed an amount of fiber above the recommendation and the women who consumed an amount of fiber below the recommendation. We did not expect this result. It is accepted that fiber stimulates butyrate production [41], so we expected that the women who consumed greater amounts of dietary fiber would have a higher abundance of bacterial butyrate-producing genes. Future work should more carefully assess the full functional potential of the gut microbiota in those who meet the fiber intake recommendation, perhaps through whole genome sequencing of the stool metagenome or through analyses of the plasma, urine, and/or stool metabolomes.

### Study Strengths and Limitations

A few limitations of our study should be acknowledged. One limitation was our modest sample size of 52 pregnant women, with only 8 of those participants meeting their fiber recommendation. However, the relatively small proportion of women who met their fiber recommendation (14.8%) is reflected in the findings of several other studies [1,9,10], emphasizing that there is an urgent need for improved guidance to help pregnant women meet their dietary fiber goals. Our data distribution was concentrated at the low end of fiber intake, so it is difficult to use this data set to detect linear relationships between fiber intake and gut microbiota composition or other health outcomes. A larger population of healthy pregnant women with higher levels of fiber intake is necessary to determine if there is an association between dietary fiber and gut microbiota composition during pregnancy.

Additionally, this study assessed dietary fiber intake using the five-factor screener [22,23], which estimates intake based on standardized portion sizes and food frequency rather than individual-specific portion sizes. Since portion sizes can vary widely, this method may have led to some overestimation or underestimation of fiber intake. While the screener is validated for population-level assessment, future research could improve accuracy by incorporating a more detailed dietary assessment method. This study did not collect data on total caloric intake, which may have influenced the assessment of fiber intake and its associations with microbiota outcomes. Due to the small number of participants who met the fiber recommendations and the focus on comparing those who met to those who did not meet the recommendations, our study did not analyze fiber intake by quartiles. Future research with a larger sample size would allow for a more comprehensive evaluation. The measure of butyrate used herein was based on gene abundance rather than a functional assay; thus, the results indicate that butyrate production capacity was similar, but the results do not reflect gene expression or the functional output of the genes. Further, this study did not analyze the correlation between *buk* or *but* abundance and specific taxa and fiber intake or compare Ct levels among microbiome-dominant groups due to sample size and data limitations. Future studies with larger cohorts and detailed microbial analysis could explore these relationships further. This study did not collect data on the specific types of fiber consumed, which limited our ability to assess their individual effects on gut microbiota.

Our study had several strengths, including that the participants were drawn from a sample representative of the population of Michigan’s lower peninsula. Additionally, the survey we used to calculate fiber and vegetable intake is validated and has been used widely with adults [22,42,43]. Another strength is that a designed, targeted assay was used to measure the abundance of the genes responsible for the last step of the butyrate production process in bacteria.

## 5. Conclusions

In conclusion, 84.7% and 92.3% of the pregnant women in this Michigan cohort consumed an amount of dietary fiber and vegetables, respectively, that is below the recommendation put in place by the Dietary Guidelines for Americans 2020–2025. The women who met the pregnancy fiber recommendation had gut microbiotas that were more diverse; however, neither the membership nor community structure differed between those who met and those who did not meet the pregnancy fiber recommendation.

## Figures and Tables

**Table 1:** Participant characteristics for women who met dietary fiber recommendations during pregnancy versus those who did not.

Participant Characteristic		Met Recommendation (*n* = 8)	Did Not Meet Recommendation (*n* = 44)	*p*-Value
Age (years)	Mean (SD) (overall)	31.4 (5.1)		
	18–27 (*n* = 10)	25% (2)	18% (8)	0.07
	28–37 (*n* = 38)	50% (4)	77% (34)	
	38–47 (*n* = 3)	12.5% (1)	4.5% (2)	
	48–57 (*n* = 1)	12.5% (1)	0% (0)	
BMI Category ^a^	Mean (SD) (overall)		27.28 (7.28)	
	Normal (*n* = 25)	33% (2)	55% (23)	0.53
	Overweight (*n* = 9)	33% (2)	28% (7)	
	Obese (*n* = 13)	33% (2)	17% (12)	
Race ^b^				
	White (*n* = 41)	67% (4)	92.5% (37)	0.02 *
	Black (*n* = 3)	33% (2)	2.5% (1)	
	Other (*n* = 2)	0% (0)	5% (2)	
Ever Smoked ^c^				
	Yes (*n* = 8)	0% (0)	21% (8)	0.52
	No (*n* = 37)	100% (6)	79% (31)	
Annual Household Income ^a^				
	<USD 50,000 (*n* = 28)	50% (3)	59.5% (25)	0.49
	USD 50,000–USD 75,000 (n = 7)	0% (0)	16.6% (7)	
	USD 75,000–USD 100,000 (*n* = 9)	33% (2)	16.6% (7)	
	>USD 100,000 (*n* = 4)	17% (1)	7% (3)	
Education				
	Did not finish high school (*n* = 1)	0% (0)	2% (1)	0.47
	High school graduate or GED (*n* = 7)	25% (2)	11% (5)	
	Some college, associate degree, or trade school (*n* = 17)	12.5% (1)	36% (16)	
	Bachelor’s degree or more (*n* = 27)	62.5% (5)	50% (22)	

* *p*-value < 0.05. ^a^: missing *n* = 4, ^b^: missing *n* = 6, ^c^: missing *n* = 7.

## Data Availability

The data presented in this study are available on request from the corresponding author. The data are not publicly available due to the sample size and consortium requirements.

## Supplementary Materials

The following supporting information can be downloaded at https://www.mdpi.com/article/10.3390/nu17050773/s1: Table S1: Bacterial counts of taxa with an average abundance of greater than or equal to 1% relative abundance were included in the negative binomial analysis. False discovery rate (FDR)-adjusted *p*-values are reported for bacterial taxa count comparisons between women who met their fiber recommendation during pregnancy (Yes, *n* = 8) and those who had not (No, *n* = 44).
