# Supplementary material for: The Relationship Between Fiber Intake and Gut Bacterial Diversity and Composition During the Third Trimester of Pregnancy
Source: Nutrients. 2025 Feb 22;17(5):773. doi: 10.3390/nu17050773 (PMC11901921; doi:10.3390/nu17050773)
Supplement: Supplementary file 1 [file nutrients-17-00773-s001.zip › nutrients-3477015-supplementary.pdf]

Supplemental Table S1. Bacterial counts of taxa with an average abundance of greater than or equal to 1% relative abundance were included in negative binomial analysis. False discovery rate (FDR)-adjusted *p*-values are reported for bacterial taxa count comparisons between women who had met their fiber recommendation during pregnancy (Yes, *n* = 8) and those who had not (No, *n* = 44).

Taxa	Overall (Median IQR)	No	Yes	*p*–Value
Lachnospiraceae unclassified	1080 (709–1373)	1044.5 (661.25–1367.5)	1143 (1058–1309.5)	0.9406
Ruminococcaceae unclassified	863 (223–1286)	847.5 (181–1280.75)	847.5 (181–1280.75)	0.9406
Bacteroides	775 (174–1754)	771.5 (174–1750.5)	1559 (288–1729)	0.9406
Faecalibacterium	480 (314–680)	457 (315.75–675)	566 (326.5–769.5)	0.9406
Clostridiales unclassified	66 (15–347)	57 (12.5–336)	66 (52–345.5)	0.9406
Prevotella	149 (5–3832)	346 (5.25–4902.75)	13 (6–780.5)	0.5839
Phocaeicola	855 (387–2102)	862.5 (392–1963.5)	590 (329.5–2354.5)	0.9406
Blautia	175 (111–976)	172.5 (113.5–1019.5)	397 (115.5–792)	0.9406
Bacteria unclassified	23 (2–122)	23 (3–67.75)	83 (24–705.5)	0.5839
Bifidobacterium	182 (53–386)	133.5 (49.5–377)	235 (174.5–455)	0.9406
Alistipes	162 (41–603)	172 (28.25–599)	150 (76–421)	0.9406
Parabacteroides	212 (77–563)	188.5 (79.5–576.5)	224 (79.5–323)	0.9406
Roseburia	100 (20–238)	103 (19.25–219.25)	31.5 (378–132)	0.9406
Phascolarcto–bacterium	48 (1–491)	15.5 (0.25–472.75)	132 (28–508)	<0.0001 *
Megasphaera	1 (0–101)	1 (0–60.75)	4 (0–101)	0.9406
Akkermansia	22 (1–372)	9 (0.25–351.25)	58 (34–227)	0.9406
Prevotellamassilia	0 (0–0)	0 (0–0)	0 (0–2.5)	<0.0001 *
Massiliprevotella	0 (0–4)	0 (0–2.75)	0 (0–28.5)	0.9406

* = *p*-value < 0.05.
